# International External Quality Assessment Study for Molecular Detection of Lassa Virus

**DOI:** 10.1371/journal.pntd.0003793

**Published:** 2015-05-21

**Authors:** Sergejs Nikisins, Toni Rieger, Pranav Patel, Rolf Müller, Stephan Günther, Matthias Niedrig

**Affiliations:** 1 Highly Pathogenic Viruses (ZBS 1), Centre for Biological Threats and Special Pathogens, Robert Koch Institute, Berlin, Germany; 2 European Public Health Microbiology Training Programme (EUPHEM), European Centre for Disease Prevention and Control (ECDC), Stockholm, Sweden; 3 Bernhard-Nocht-Institute for Tropical Medicine, WHO Collaborating Centre for Arboviruses and Hemorrhagic Fever Reference and Research, Hamburg, Germany; 4 Biomatrica, San Diego, California, United States of America; Aix Marseille University, Institute of Research for Development, and EHESP School of Public Health, FRANCE

## Abstract

Lassa virus (LASV) is a causative agent of hemorrhagic fever in West Africa. In recent years, it has been imported several times to Europe and North America. The method of choice for early detection of LASV in blood is RT-PCR. Therefore, the European Network for Diagnostics of ‘Imported’ Viral Diseases (ENIVD) performed an external quality assessment (EQA) study for molecular detection of LASV. A proficiency panel of 13 samples containing various concentrations of inactivated LASV strains Josiah, Lib-1580/121, CSF, or AV was prepared. Samples containing the LASV-related lymphocytic choriomeningitis virus (LCMV) and negative sera were included as specificity controls. Twenty-four laboratories from 17 countries (13 European, one African, one Asian, two American countries) participated in the study. Thirteen laboratories (54%) reported correct results, 4 (17%) laboratories reported 1 to 2 false-negative results, and 7 (29%) laboratories reported 3 to 5 false-negative results. This EQA study indicates that most participating laboratories have a good or acceptable performance in molecular detection of LASV. However, several laboratories need to review and improve their diagnostic procedures.

## Author Summary

A proficiency test panel for molecular diagnostic of Lassa virus provides objective evidence of testing quality of International diagnostic laboratories. Since there are no commercial assays available, it is very important to assess the quality of diagnostic test used as well as evaluate detection sensitivity and specificity performance. Participating laboratories have received samples containing different inactivated Lassa virus strains as well as two negative controls. Participants were asked to provide information on diagnostic test procedure and protocols used for analysis of samples of Lassa virus External Quality Assessment (EQA). Based on received information we were able to compare and evaluate the quality of diagnostic profile and facilitate further improvement. Participating laboratories may use results of Lassa virus EQA to become accredited for Lassa virus molecular diagnostic. Since different Lassa virus strains are not available for most of the laboratories, participants achieved very advanced training for diagnostic of rare and imported viruses.

## Introduction

Lassa fever was first described in 1969 as the cause of a nosocomial outbreak of hemorrhagic fever in Nigeria [1]. Lassa fever is an acute viral infection associated with a wide spectrum of disease manifestations, which range from mild courses to multiorgan failure [1–3]. The etiologic agent of Lassa fever is Lassa virus (LASV, family *Arenaviridae*, genus *Arenavirus*) [4]. The natural host of LASV is the small rodent *Mastomys natalensis*, which lives close to human settlements [5]. The rodents can become chronically infected at birth and excrete infectious virus in urine and other body fluids, with subsequent transmission to humans [6]. There is evidence of human-to-human transmission in both hospital and community settings [7]. The fact that LASV may be transmitted from human to human gives rise to nosocomial or community-based outbreaks. LASV is endemic in the countries of Nigeria, Liberia, Sierra Leone, and Guinea [8, 9] and was detected in Mali [10, 11]. Seroepidemiological studies and imported cases of Lassa fever indicate that arenaviruses circulate somewhere in the region comprising Côte d’Ivoire and Burkina Faso [12]. The annual incidence is estimated at 300,000 cases, with 5,000 fatalities per year [13, 14]. Additionally, LASV has been introduced several times into Europe, Japan, and North America. Among the hemorrhagic fever viruses of risk group 4 (such as Crimean-Congo hemorrhagic fever, Ebola, and Marburg virus), LASV has been most frequently imported [15]. The virus usually is imported by returning travelers [16, 17]. Within Europe, LASV infections have been imported to Germany [18, 19], The Netherlands [20] and the United Kingdom [21].

Laboratory testing is required to establish a diagnosis, as Lassa fever can hardly be distinguished from other febrile diseases based on clinical symptoms [14, 22]. A suspected case must be rapidly ruled out or verified to facilitate appropriate case management, including treatment, the implementation of isolation measures, or the tracking of contact persons [18].

The method of choice for early detection of LASV in blood is reverse transcription (RT)-PCR [23–29]. However, the high degree of genetic variability of the virus poses a problem with the design of RT-PCR assays for the reliable detection of all virus strains [30]. The performance of the different techniques applied for molecular diagnosis of LASV may vary between laboratories. External quality assessment (EQA) studies for LASV molecular diagnostics have not been performed since 2004 [31]. An EQA study allows the participating laboratories to monitor the quality of their diagnostics and to identify problems with particular diagnostic assays. For these reasons, an EQA study for the molecular diagnosis of LASV was conducted by the European Network for Diagnostics of ‘Imported’ Viral Diseases (ENIVD) (http://www.enivd.org) in 2013. ENIVD is concerned with the development of laboratory diagnostic capacities for imported virus infections, quality control, standardization of laboratory procedures, and training of laboratory staff [32]. Based on the results of this study, the quality of LASV diagnostics may be improved.

## Materials and Methods

### Call for participation

Twenty-eight laboratories involved in diagnostics of viral hemorrhagic fevers were invited to participate in this study. Invitees were selected from the register of ENIVD network members and from the list of national and regional reference laboratories for rare, emerging, and dangerous viruses. The participation in the study was free of charge. Participants permitted publication of the results in a comparative and anonymous manner. This EQA was coordinated by ENIVD according to the established procedures of the network [33–35].

### Sample preparation

The proficiency test panel included 13 LASV preparations derived from culture supernatants of Vero E6 cells (ATCC—American Type Culture Collection) infected with 4 different LASV strains. Virus in cell culture supernatant was inactivated by heat (1 h at 60°C) followed by gamma irradiation (25 kilo gray). The test panel consisted of six samples of LASV strain Josiah from Sierra Leone, obtained by serial 10-fold dilution of cell culture supernatant (1:10 to 1:10^6^), three samples of LASV strain Lib-1580/121 from Liberia (dilutions 1:10^3^ to 1:10^5^), LASV strain CSF from Nigeria (dilution 1:10^3^), and LASV strain AV from Cote d’Ivoire or Burkina Faso (dilution 1:10^3^). The samples were freeze-dried in 3% mannitol based formulation using an EPSILON 2-6D Pilot Freeze Dryer (Martin Christ GmbH, Osterode am Harz, Germany). In addition, we included one sample containing LASV strain Josiah at a dilution of 1:10^4^ (sample #14) that was prepared with a new dry stabilizer method (Biomatrica, Inc., San Diego, CA, USA), and one sample containing LASV strain Lib-1580/121 at dilution of 1:10^4^ (sample #6) that was prepared using a liquid stabilizer (Biomatrica). A sample containing lymphocytic choriomeningitis virus (LCMV), the prototype member of the family *Arenaviridae*, as well as two negative control sera were included in the test panel as specificity controls.

After lyophilized sample preparation, the samples were tested and quantified by an in-house real-time PCR assay for quality control purpose. The assay was performed by employing 12.5 pmol of forward primer LaV F2 (CCACCATYTTRTgCATRTgCCA), 13 pmol of reverse primer LaV R (gCACATgTNTCHTAYAgYATggAYCA) and 5 pmol of probe LaV TM (FAM-AARTggggYCCDATgATgTgYCCWTT-BBQ). The real-time PCR assay was carried out in one-step format on ABI 7500 real-time PCR system using the AgPath-ID One-Step RT-PCR Kit according to manufacturer´s instruction. Plasmid standards were used for the quantification of the genome copies of Lassa virus RNA.

### Ethics statement

The EQA was performed according to National Ethical regulations.

### Validation and dispatch of the panel sets

Before dispatching the panel, samples were sent to the reference laboratory for testing the quality and obtaining the reference results. Reference laboratory used RT-PCR protocol described by Ölschläger et al., 2010 [27]. Samples were resuspended in 100 μl of water and the RNA was extracted using the QIAamp viral RNA kit (Qiagen, Hilden, Germany). The presence of LASV or LCMV RNA in the samples was ascertained by RT-PCR and sequencing. The number of LASV genome copies present in these samples was determined by qRT-PCR. Samples were shipped by regular mail at ambient temperature. Participating laboratories were instructed to resuspend the samples in 100 μl of water and to analyze the material like serum samples potentially containing LASV using their routine nucleic acid detection assays. The EQA panel was accompanied by documentation including instructions and an evaluation form for results. Participants were asked to report the assay protocol, the result for each sample, the LASV strain identified, the number of genome copies as well as any problem encountered.

### Evaluation of the results

To guarantee anonymous data evaluation and reporting, each participating laboratory was coded with an identifier. The results were scored according to detection rate and specificity as in previous EQA studies of ENIVD [33–35]. We assigned one point for correct results; false-negative, false-positive, and indeterminate results did not count. Results were classified as “good”—when all results were correct; “acceptable”—when 1 to 2 results were incorrect; and “need for improvement”—when more than 2 results were incorrect. Results for the sample containing LCMV (sample #3) were not included in the score, as verification of the sequence was optional. In addition, we excluded from scoring the sample containing LASV strain Josiah at a dilution of 1:10^6^ (sample #15) as this concentration is likely to be below the 95%-detection limit of most assays. Thus, obtaining a positive or negative result becomes a matter of chance. Each laboratory received the complete summary of the results in an anonymous way, by which only the own laboratory was recognizable.

## Results

Twenty-four (86%) of the 28 laboratories, which received the EQA material, reported results. The 24 participating laboratories, located in 17 countries—13 European, one African, one Asian, and two American countries (Table 1). The LASV detection rate varied among laboratories and scores ranged from 9 to the maximum of 14 (Table 2). Average score for all participating laboratories was 13 points. Good results were achieved by 13 (54%) laboratories, 4 (17%) laboratories achieved acceptable results, and 7 (29%) laboratories had need for improvement. Table 3 shows that 13 (54%) laboratories correctly detected LASV in all 12 LASV samples (100% detection rate). Three (12%) participants had one false negative result (92% detection rate). Eight participants had a detection rate between 58% and 83%. None of the laboratories reported false-positive results for the negative control samples.

**Table 1:** List of participating laboratories.

Name of Participating laboratory	City, Country
Arbovirus and Imported Viral Disease Unit, Centro Nacional de Microbiologia, Instituto de Salud Carlos III	Madrid, Spain
Aristotle University of Thessaloniki, School of Medicine A, Department of Microbiology	Thessaloniki, Greece
Cantacuzino Institute Vector-Borne Diseases & Medical Entomology	Bucharest, Romania
Departamento de Microbiología, Hospital Clinic i Provincial de Barcelona (Barcelona, Spain); Dipartimento di Istologia, Microbiologia e Biotecnologie Mediche, Università di Padova	Padova, Italy
DSO National Laboratories	Singapore, Singapore
Erasmus MC Nb 1052, Dept. Viroscience	Rotterdam, The Netherlands
Institut für Mikrobiologie der Bundeswehr, Zentralbereich Diagnostik	München, Germany
Institute for Novel and Emerging Infectious Diseases, Friedrich-Loeffler-Institut);	Greifswald—Insel Riems, Germany
Institute of Microbiology and Immunology, University of Ljubljana	Ljubljana, Slovenia
Laboratoire P4 Inserm Jean Mérieux	Lyon, France
Laboratory of Virology, University Hospitals of Geneva	Geneva, Switzerland
Laboratory of Virology, National Institute for Infectious Diseases "L Spallanzani"	Rome, Italy
National Center for Epidemiology virologické odd.	Budapest, Hungary
Rare & Imported Pathogens Department, Public Health England	Porton Down, Salisbury, UK
Swedish Institute for Infectious disease control	Stockholm, Sweden
Vector Design and Immunotherapy, National Microbiology Laboratory, Public Health Agency of Canada	Winnipeg, Canada
Special Pathogens Unit, National Institute for Communicable Diseases	Sandringham, South Africa
Spiez Laboratory—Virology	Spiez, Switzerland
Spiez Laboratory—Federal Office for Civil Protection	Spiez, Switzerland
TIB MOLBIOL Syntheselabor GmbH	Berlin, Germany
Unit for Emergency Response and Biopreparedness, National Institute of Health	Lisbon, Portugal
Unité de Virologie-IRBA	Lyon, France
Viral Special Pathogens Branch, National Center for Emerging and Zoonotic Infectious Diseases, CDC	Atlanta, U.S.A
Departamento de Microbiología Hospital Clinic i Provincial de Barcelona	Barcelona, Spain

**Table 2:** Summary of the EQA study for molecular detection of LASV.

	Result according to sample no., virus strain, and dilution																
	#16	#7	#5	#1	#14^a^	#9	#15^b^	#12	#4	#10	#13	#6^a^	#2	#3^b^	#11^b^	#8^b^	
	LASV Josiah	LASV Josiah	LASV Josiah	LASV Josiah	LASV Josiah	LASV Josiah	LASV Josiah	LASV AV	LASV CSF	LASV Lib 1580	LASV Lib 1580	LASV Lib 1580	LASV Lib 1580	LCMV	Neg	Neg	
Participant number	1:10^1^	1:10^2^	1:10^3^	1:10^4^	1:10^4^	1:10^5^	1:10^6^	1:10^3^	1:10^3^	1:10^3^	1:10^4^	1:10^4^	1:10^5^	1:10^2^			Score
1	+	+	+	+	+	+	+	+	+	+	+	+	+	–	–	–	14
2	+	+	+	+	+	+	+	+	+	+	+	+	+	+	–	–	14
4	+	+	+	+	+	+	+	+	+	+	+	+	+	+	–	–	14
5	+	+	+	+	+	+	+	+	+	+	+	+	+	+	–	–	14
6	+	+	+	+	+	+	–	+	+	+	+	+	+	–	–	–	14
10	+	+	+	+	+	+	+	+	+	+	+	+	+	–	–	–	14
14	+	+	+	+	+	+	–	+	+	+	+	+	+	LASV	–	–	14
15	+	+	+	+	+	+	+	+	+	+	+	+	+	+	–	–	14
16	+	+	+	+	+	+	–	+	+	+	+	+	+	–	–	–	14
17	+	+	+	+	+	+	–	+	+	+	+	+	+	–	–	–	14
18	+	+	+	+	+	+	–	+	+	+	+	+	+	–	–	–	14
21	+	+	+	+	+	+	+	+	+	+	+	+	+	–	–	–	14
23	+	+	+	+	+	+	–	+	+	+	+	+	+	LASV	–	–	14
13	+	+	+	+	–	+	+	+	+	+	+	+	+	+	–	–	13
19	+	+	+	+	+	+/–	–	+	+	+	+	+	+	LASV	–	–	13
20	+	+	+	+	+	+	–	+	+	+	+	+	–	+	–	–	13
12	+	+	+	+	+	–	–	+	+	+	+	–	+	–	–	–	12
7a	+	+	–	+	+	+	+	+	+	+	–	+	–	LASV	–	–	11
7b	+	+	–	+	+	+	+	+	+	+	–	+	–	–	–	–	11
22	+	+	+	+	+	+	+	+	+	+	–	–	–	+	–	–	11
3	+	+	+	+	+	+	+	+	+	–	–	–	–	LASV	–	–	10
9	+	+	+	+	+	+	–	+	+	–	–	–	–	+	–	–	10
11	+	+	+	+	+	+	+	+	+	–	–	–	–	+	–	–	10
8	+	+	+	+	+	–	–	+	+	–	–	–	–	+	–	–	9

^a^ with stabilizer;

^b^ not included in score;

+, virus correctly detected;—negative result; +/–, indeterminate result

**Table 3:** LASV detection rate by participant.

Participant number	False-negative results	Detection rate, %
1, 2, 4, 5, 6, 10, 14, 15, 16, 17, 18, 21, 23	0/12	100
13, 19, 20	1/12	92
12	2/12	83
7a, 7b, 22	3/12	75
3, 9, 11	4/12	67
8	5/12	58

All participating laboratories were able to detect LASV strain Josiah at 1:10, 1:100, and 1:10^4^ dilution (Table 4). Two laboratories did not detect the Josiah strain at 1:10^3^ dilution (sample #5) but detected the 1:10^4^ to 1:10^6^ dilutions of this strain (Table 2). A mix-up between sample#5 and sample #6 is a likely explanation. LASV strains AV from Cote d’Ivoire/Burkina Faso and CSF from Nigeria were detected correctly by all laboratories. Four laboratories did not detect at all the Liberian LASV strain. Further laboratories did not detect the higher dilutions of the Liberian strain (≥ 1:10^4^).

**Table 4:** LASV detection rate by sample.

	Result according to sample no., virus strain, and dilution												
	#16	#7	#5	#1	#14^a^	#9	#15^b^	#12	#4	#10	#13	#6^a^	#2
	LASV Josiah	LASV Josiah	LASV Josiah	LASV Josiah	LASV Josiah	LASV Josiah	LASV Josiah	LASV AV	LASV CSF	LASV Lib 1580	LASV Lib 1580	LASV Lib 1580	LASV Lib 1580
	1:10^1^	1:10^2^	1:10^3^	1:10^4^	1:10^4^	1:10^5^	1:10^6^	1:10^3^	1:10^3^	1:10^3^	1:10^4^	1:10^4^	1:10^5^
False-negative results	0/24	0/24	2/24	0/24	1/24	3/24	11/24	0/24	0/24	4/24	7/24	6/24	8/24
Detection rate, %	100	100	92	100	96	88	54	100	100	84	71	75	67

^a^ with stabilizer;

^b^ not included in score

Nineteen participants used published RT-PCR protocols, two laboratories used unpublished in-house RT-PCR assays, one laboratory used a combination of real-time and conventional RT-PCR, and two laboratories did not provide information about the protocol. Three laboratories confirmed their results by using a second published protocol. Table 5 shows that 11 (50%) laboratories used the protocol published by Ölschläger et al., 2010 [26]. These 11 laboratories reported nine false-negative results (detection rate of 93% for this protocol). Seven laboratories using the Ölschläger et al. protocol did not report any false-negative result. Six participants (27%) used the protocol of Vieth et al., 2007 [29] (Table 5). They reported 12 false-negative results (detection rate of 83% for this protocol). Two laboratories using this protocol did not report any false-negative results. Three other protocols (Demby et al., 1994 [23]; Coulibaly N’Golo et al, 2011 [28]; Drosten et al., 2002 [27]) were used by one or two laboratories (Table 5). In the supplementary file (S1 Fig) we are showing partial alignments of the S-segment sequences of LASV strains used in the EQA with position of some published primers: (A) forward primer sequence and position on S-segment alignment; (B) reverse primer sequence and position on S-segment alignment. In comparison to Demby et al., Lassa RT-PCR assay described by Ölschläger et al. had just modified reverse primer in order to detect all described Lassa virus strains.

**Table 5:** Summary of the published protocols used by the participating laboratories.

Protocol and reference	PCR method	Target gene	No. of participants using protocol	False- negative results	Detection rate, %	Number of participants with correct results (%)
Ölschläger et al., 2010 [26]	One-step RT-PCR	GPC gene	11	9/132	93.2	7 (63.6)
Vieth et al., 2007 [29]	RT-PCR	L-gene	6	12/72	83.3	2 (33.3)
Demby et al., 1994 [23]	RT-PCR	GPC gene	2	1/24	95.8	1 (50)
Drosten et al., 2002 [27]	Sybr qRT-PCR	GPC gene	2	4/24	83.3	1 (50)
Coulibaly N’Golo et al., 2011 [28]	RT-PCR	L-gene/ GPC gene	1	0/12	100.0	1 (100)

Ten laboratories (42%) reported the presence of LCMV in sample #3. In addition, 9 (37%) laboratories reported this sample as negative, which is also considered a correct result because this EQA was conducted to test for the ability to detect LASV. Five laboratories (21%) reported the sample containing LCMV as positive for LASV. These laboratories used protocols for detection of Old World arenaviruses, including LCMV. This underlines the relevance of sequencing the diagnostic PCR products when using pan-virus family detection assays.

### Conclusions

This EQA study indicates that most participating laboratories, located in various countries around the world, have a good or acceptable performance in molecular detection of LASV. However, several laboratories need to improve their performance, in particular with respect to detection of the Liberian strain. The data allow the participating laboratories to identify the weakness in their diagnostic procedures and to review and improve their protocols.

One published protocol has achieved 100% detection rate reported by single participant. However, the reference laboratory recommends Ölschläger et al., 2010 published protocol for LASV detection as most commonly used with good detection rate and ability to detect all described Lassa virus strains. The main aim of this EQA study was not to compare published protocols, rather to give chance to participating laboratories to evaluate their testing performance and provide practical exercise for molecular detection of LASV. There should be a follow-up EQA for molecular detection of LASV to evaluate a possible improvement.

## Supporting Information

S1 Fig Partial alignments of the S-Segment sequences of LASV strains used in the EQA with position of some published primers. (TIF)
