# Supplementary figures and images for: International External Quality Assessment Study for Molecular Detection of Lassa Virus
Source: PLoS Negl Trop Dis. 2015 May 21;9(5):e0003793. doi: 10.1371/journal.pntd.0003793 (PMC4440764; doi:10.1371/journal.pntd.0003793)

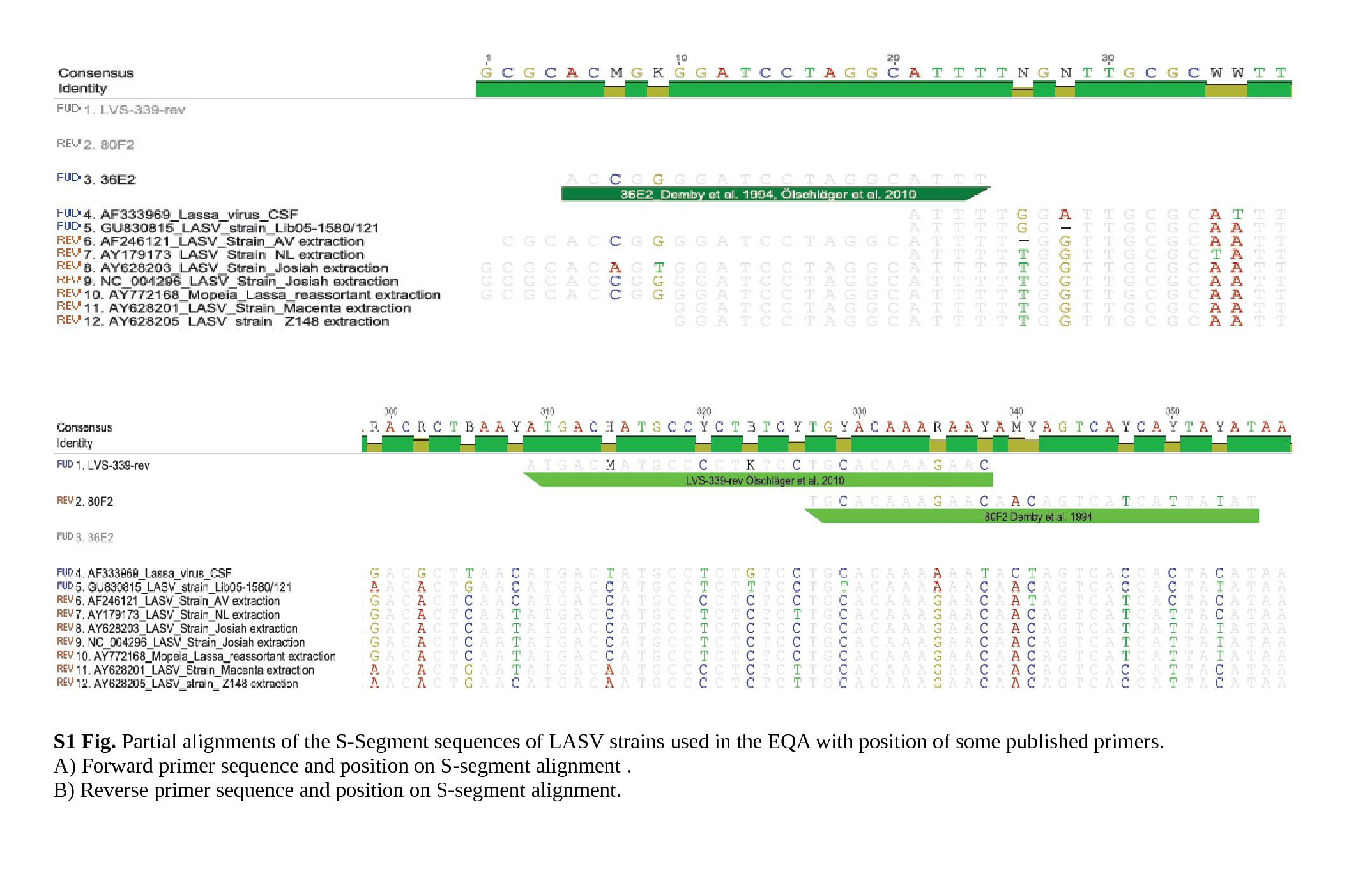

Supplement: S1 Fig — (TIF) [file pntd.0003793.s001.tif]
